# Evaluation of the efficacy of commercial live vaccines against the local Thai QX field strain for the protection of specific pathogen-free chicks

**DOI:** 10.14202/vetworld.2024.771-777

**Published:** 2024-04-07

**Authors:** Thotsapol Thomrongsuwannakij, Doan Hoang Phu, Niwat Chansiripornchai

**Affiliations:** 1 Akkhraratchakumari Veterinary College, Walailak University, Nakorn Si Thammarat, Thailand; 2 Centre for One Health, Walailak University, Nakhon Si Thammarat, Thailand; 3 Department of Infectious Diseases and Veterinary Public Health, Faculty of Animal Science and Veterinary Medicine, Nong Lam University, Ho Chi Minh City, Vietnam; 4 Avian Health Research Unit, Department of Veterinary Medicine, Faculty of Veterinary Science, Chulalongkorn University, Bangkok, Thailand

**Keywords:** avian infectious bronchitis, chicken, efficacy, QX-like strain, Thailand, vaccine

## Abstract

**Background and Aim:**

The high prevalence of QX-like variant among Thai isolates poses a significant threat to poultry production. In this study, we evaluated the protective efficacy of commercially available heterologous infectious bronchitis virus (IBV) vaccines against the local Thai QX-like strain in specific-pathogen-free (SPF) chicks from Thailand.

**Materials and Methods:**

The experiment involved 100 SPF chicks divided into 4 arms. Arms I and II received the TAbic IB VAR (233A) and Ibird (1/96) vaccines, respectively, on day 1. After 10 days, both arms received the H120 vaccine. Arms III and IV were non-vaccinated positive and negative controls. Challenge infection was local Thai QX-like virus on birds of Arms I, II, and III, and negative control of Arm IV. Clinical signs of infectious bronchitis (IB) and IBV detection using reverse transcription polymerase chain reaction were assessed at 2, 4, and 6 days post-challenge (dpc). At 6 dpc, the birds were humanely euthanized for post-mortem examination with the ciliostasis test and histopathological analysis of the tracheas, lungs, and kidneys.

**Results:**

Virus shedding started at 4 dpc (33.3% positive) and reached 100% positivity at 6 dpc with obvious clinical respiratory symptoms in non-vaccinated-challenged birds. No detection of IBV in vaccinated-challenged arms. Ciliary activity scores were significantly lower in non-vaccinated-challenged birds at 23.64 (standard deviation [SD] ± 1.74) and 96.50 (SD ± 1.91) and 95.64 (SD ± 1.77), respectively (p = 0.05) than in vaccinated-challenged birds. The most remarkable histopathological changes were observed in non-vaccinated-challenged birds, with moderately severe changes in the trachea, lungs, and kidneys. On the other hand, birds in vaccinated-challenged arms showed no significant changes.

**Conclusion:**

This study demonstrated the efficacy of TAbic IB VAR (233A) or Ibird (1/96) vaccine combined with a Massachusetts serotype vaccine (H120) against the local Thai QX-like strain in SPF chicks, contributing valuable insights to the selection of suitable commercially available vaccines to combat the prevalent local QX-like strains in Thailand.

## Introduction

Avian infectious bronchitis (IB) is a highly contagious acute respiratory tract disease caused by the infectious bronchitis virus (IBV) that affects chickens and other avian species. IBV is a single-stranded RNA virus classified as a gammacoronavirus with a widespread worldwide distribution [1]. Rapid transmission occurs within chicken flocks through inhalation or direct contact with infected birds, contaminated waste, equipment, or other fomites [2]. IBV initiates infection by causing respiratory tract lesions, leading to severe infections in other organs, such as kidneys and reproductive tract [3]. The incidence of IB is almost always 100% in birds [4]; however, mortality can vary depending on factors such as age (i.e., young birds), the immune status of the birds, viral strain, and the presence of secondary opportunistic bacterial infections [1]. The impact of IB on chicken production systems leads to economic losses, reduced weight gain and feed efficiency in broilers, and reduced egg production and egg-shell quality in layer and breeder chickens [5].

Within the wide diversity of serotype and genotype compositions among IBV strains, the QX-like strain was initially isolated in Chinese chickens in 1996 [6]. The latest IBV nomenclature using a phylogeny-based classification system for nucleotide sequences of the S1 gene from IBV strains around the world has identified 32 distinct viral lineages grouped into six genotypes. Among the 1652 IBV strains used for IBV nomenclature, the QX virus with the most significant number (33.0%) was collected and classified under the new nomenclature as the GI-19 lineage [7]. Numerous reports have described the prominent QX-like strain in avian IB globally, including China [8], Japan [9], South Korea [10], Europe [11], the Middle East [12], and various African countries [13, 14]. The QX-like IBV outbreak in Thai broiler flocks was first documented in 2009 [15]. Subsequent investigations of Thai QX-like IBV cases revealed the presence of a recombinant virus arising from the recombination of the Thai QX-like virus with another strain of Chinese IBV [16]. A previous report indicated a high prevalence of QX-like variant (62.5%) among Thai isolates [17]. Exposure to IB QX within the initial week of life likely impedes the final stage of post-embryonic development, manifesting as an obstruction in the posterior section of the oviduct, driven by inflammation and subsequent strictures within the walls of the cloaca or the oviduct’s entry, resulting in a consequential cascade, including fluid accumulation and oviduct dilatation, causing inflammation and false layer syndrome [18, 19]. These conditions significantly threaten the poultry production system and highlight the complex challenges associated with the QX-like IBV strain in poultry management.

Vaccination is the cornerstone for preventing and controlling IB in poultry farming [20]. However, due to the effects of genetic changes and genome recombination, the efficacy of vaccination against recently emerging isolates has been limited [21]. The lack of cross-protection stemming from these modifications makes immunization more difficult to control and prevent IB [22]. A recent study using a heterologous vaccine strategy combining IBV serotypes (Massachusetts and 793/B) showed 81% protection against the QX-like strain [23]. Vaccines targeting strains such as M41, H120, Ma5, Connecticut (Conn), 4/91, and local DLD strains have seen widespread application in Thailand [16]. Therefore, there is a critical need to identify a suitable vaccine from the commercially available IB vaccines in Thailand against local QX-like strains, which are currently predominant in Thailand.

The objective of this study was to evaluate the protective efficacy of applying heterologous IBV vaccines in conjunction with a Massachusetts serotype vaccine (H120), specifically 793/B vaccines, including TAbic IB VAR (233A) and Ibird (1/96). This combined vaccination strategy was implemented at 1 and 10 days of age in specific-pathogen-free (SPF) chicks, aiming to assess protection against the local Thai QX-like (THA80151) strain challenge virus.

## Materials and Methods

### Ethical approval

The study protocol was in accordance with Chulalongkorn University’s guidelines and legislative regulations on animal care and use for scientific purposes. This study was approved by the Chulalongkorn University Animal Care and Use Committee (Ref. No. CU-ACUC/1973016).

### Study period and location

The whole experiment was performed from April 2020 to December 2020, and animal trials were started from June 2020 to August 2020 at Avian Health Research Unit, Faculty of Veterinary Science, Chulalongkorn University, Nakorn Pathom campus, Thailand.

### Bird selection

One hundred chickens were employed in this study, all of which were sourced as SPF chicks hatched from SPF eggs within SPF flocks. The chickens were reared in negative-pressure isolators throughout the research period and monitored daily. *Ad libitum* access to age-appropriate commercial feed and tap water was ensured during the study.

### Vaccine and challenge virus

Commercial live vaccines readily available in Thailand were utilized in the study. TAbic IB VAR (233A) by Phibro Animal Health Corporation (New York, USA), Ibird (1/96) by Ceva Sante Animale (Libourne, France), and Massachusetts serotype vaccine (H120) by Boehringer Ingelheim (Ingelheim am Rhein, Germany) were used. All vaccines were administered intraocularly to chickens according to the doses recommended by the manufacturer.

We used a local Thai QX-like strain (THA80151), as described in a previous study by Pohuang *et al*. [15]. The strain was isolated from the trachea of a 20-day-old broiler. We compared the S1 gene sequence of THA80151 (accession number: FJ156075) with published IBV sequences in the GenBank database, which revealed a close relationship with Chinese QX IBV. The concentration of IBV was determined using Reed and Muench’s egg infective dose at 50% (EID_50_) method [24]. On the basis of local experience, the challenge dose was approximately 1.5 × 10^4^ EID_50_/chick, and the virus was administered through the oculo-nasal route.

### Experimental design

**Figure-1:**
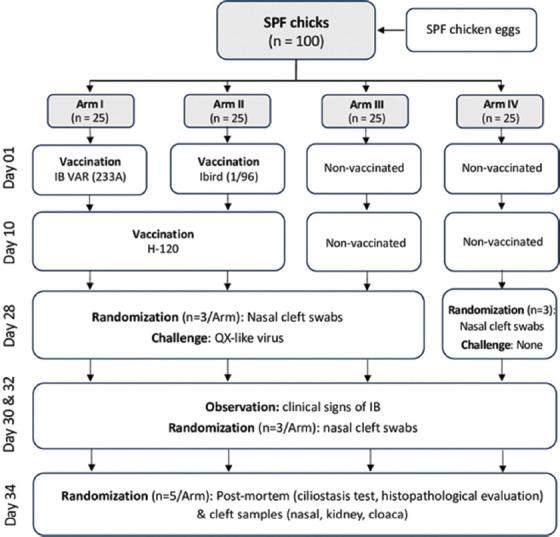
Experimental design of vaccine efficacy and pathogenicity against local Thai QX-like infectious bronchitis virus strain.

One hundred 1-day-old SPF chicks were distributed across four arms, each containing 25 birds. At 1-day of age, chicks in Arm I received TAbic IB VAR (233A) vaccination, while chicks in Arm II received Ibird (1/96) vaccination. At 10 days of age, they were further vaccinated with the Massachusetts serotype vaccine (H120). Arms III and IV were designated as positive and negative controls, respectively, without vaccination. Nasal cleft swabs were randomly obtained from five birds on day 28 to establish a baseline for QX-like IBV viral RNA detection before challenge with the local Thai QX-like virus. Challenge infections were administered to birds in Arms I, II, and III; while Arm IV served as the negative control without the local Thai QX-like virus challenge. Clinical signs of IB (i.e., gasping, coughing, tracheal rales, and nasal discharge) were observed 2 days post-challenge (dpc) (day 30) and 4 dpc (day 32). Three birds were randomly selected for nasal cleft swab collection for QX-like IBV viral RNA detection. At 6 dpc (day 34), nasal cleft swabs were collected from five randomly chosen birds for viral RNA detection of QX-like IBV. These birds were humanely euthanized for post-mortem examination to assess further ciliostasis tests and histopathological lesions of the tracheas, lungs, and kidneys. Another set of nasal cleft swabs was collected for virus isolation as a backup. The details of the experimental design are illustrated in Figure-1.

### Ciliostasis test

The ciliostasis test was used to assess the protection against the challenge. At 6 dpc, five randomly selected chickens were humanely euthanized, and their tracheas were carefully removed for examination of ciliary activity. The ciliostasis test was conducted on ten tracheal rings per chicken, comprising three tracheal rings from the upper part, four tracheal rings from the middle part, and three tracheal rings from the lower part. Each tracheal ring was examined using a light microscope at low magnification (Olympus, Center Valley, PA, USA). Ciliary movement was evaluated, ranging from 0% (complete ciliostasis) to 100% (vigorous ciliary movement) [25, 26]. We calculated the average score of ciliary activity across ten rings for each trachea for every individual chicken. Subsequently, average scores for each arm were determined. Protection was achieved when the mean score of the tracheal rings was at least 50%. The vaccine was considered to be efficacious when 80% of the birds were protected in each group [27].

### Histopathology

Histopathological evaluation was performed to examine lesions on necropsy-sampling tissues of five randomly selected birds at 6 dpc. The cranial parts of the trachea, lung, and left kidney were collected, fixed in 10% buffered formalin, embedded in paraffin, sectioned, and stained with hematoxylin and eosin for histopathological examination. In the trachea, deciliation, degeneration, and hyperplasia of the epithelia were observed, a decrease in mucous cells, infiltration of heterophils and lymphocytes, and edema. Lungs were examined for lymphoid and heterophilic infiltration. The kidneys were examined for epithelial deciliation, degeneration and hyperplasia, ductotubular dilation, heterophil and lymphoid infiltration, epithelial regeneration, lymphoid nodules, and fibroblastic proliferation. Histopathological lesion scores were measured using a scoring range: 0 = no change, 1 = mild, 2 = moderate, and 3 = severe [28]. We calculated the average score for every chicken examined and determined the average scores for each study arm.

### IBV detection

Tracheal cleft swabs were collected from randomly selected birds at 2, 4, and 6 dpc. The swabs were transported in a cool box to the laboratory within 4–6 h. The tracheal cleft swabs were cut using sterilized scissors, transferred in 1.5 mL microtube containing 800 μL of 10% sterile phosphate-buffered saline (PBS) and vortexed for 10 s, while the kidney and cloacal samples were homogenized using 10% PBS and centrifuged at 1,800× *g* for 10 min. Subsequently, the supernatant (200 μL) was collected for RNA extraction. RNA was extracted using a PureLinkTM Viral RNA/DNA Mini kit (Invitrogen, Thermo Fisher Scientific, Waltham, MA, USA) according to the manufacturer’s instructions. Primers were designed as described in a previous study [26] based on the differentiation between the challenge virus IBV (QX-like THA80151) and vaccine strains (IB H120 and IB 793/B). The primer sequences were as follows: F1547- TAATGAAACTGGTTCTCAGCC and R1691-GCGGTACTATTTGCTTAATAA. Reverse-transcription polymerase chain reaction (RT-PCR) was performed in a one-step using the AccessQuick RT-PCR System (Promega, Madison, WI, USA) at 48°C for 45 min, followed by heating at 94°C for 5 min. The amplification consisted of 35 cycles involving denaturation at 94°C for 60 s, annealing at 56°C for 30 s, and extension at 72°C for 60 s. PCR amplicons were visualized through 1.2% agarose gel electrophoresis, stained with ethidium bromide (0.5 μg/mL), and read by an ultraviolet transilluminator. The final extension occurred at 72°C for 10 min.

### Statistical analysis

All statistical analyses were performed using Statistical Package R software. Descriptive analyses were performed using means and standard deviations (SDs). Significant differences in the histopathological and ciliostasis scores among study arms were examined using non-parametric Kruskal–Wallis test after checking for normality of the distribution using Shapiro–Wilk normality test. In addition, the “ggplot2” package was applied to visualize the results of histopathological and ciliostasis scores

## Results

### Clinical signs, gross lesions, mortality after challenge

Clinical signs were exclusively observed in chickens inoculated with the QX-like strain (Arm III), which manifested as early as 4 dpc and continued until 6 dpc. At 4 dpc, most of the birds in this arm showed mild signs of depression. At 6 dpc, however, all birds displayed obvious clinical symptoms, including respiratory distress characterized by sneezing, nasal discharge, and conjunctivitis. Necropsy findings revealed tracheitis and swelling of kidneys in these birds. No clinical signs or gross lesions were observed on necropsy of chickens in arms I, II and IV. In addition, there were no cases of mortality among birds in the four study arms throughout the experimental trial period.

### IBV detection by RT-PCR

No IBV was detected across the tracheal cleft swabs of all four study arms on the challenge day and at 2 dpc. At 4 dpc, only one out of three randomized birds (33.3%) in Arm III (non-vaccinated, QX-like virus-challenged) tested positive for IBV. At 6 dpc, all tracheal samples randomly selected from birds in Arm III were positive (5/5, 100%), whereas all kidney and cloacal samples were negative. All RT-PCR tests showed negative results in the other arms, including Arm I (TAbic IB VAR [233A] + H120 vaccines), Arm II (Ibird (1/96) + H120 vaccine), and Arm IV (non-vaccinated, non-challenged). Table-1 presents the detailed findings of IBV detection.

**Table-1:** IBV detection using RT-PCR at 2 dpc, 4 dpc and 6 dpc.

Study arms	Vaccination	Challenge	Challenge day	2 dpc	4 dpc	6 dpc		
			
			Nasal cleft swab	Nasal cleft swab	Nasal cleft swab	Nasal cleft swab	Kidney sample	Cloaca sample
I	IB VAR (233A) + H120	QX-like virus (THA80151)	0/3	0/3	0/3	0/5	0/5	0/5
II	Ibird (1/96) + H120	QX-like virus (THA80151)	0/3	0/3	0/3	0/5	0/5	0/5
III	Non-vaccinated	QX-like virus (THA80151)	0/3	0/3	1/3	5/5	0/5	0/5
IV	Non-vaccinated	Non-challenged	0/3	0/3	0/3	0/5	0/5	0/5

IBV=Infectious bronchitis virus, IB=Infectious bronchitis, RT-PCR=Reverse-transcription polymerase chain reaction, dpc=Days post-challenge

### Tracheal ciliary activity

**Figure-2:**
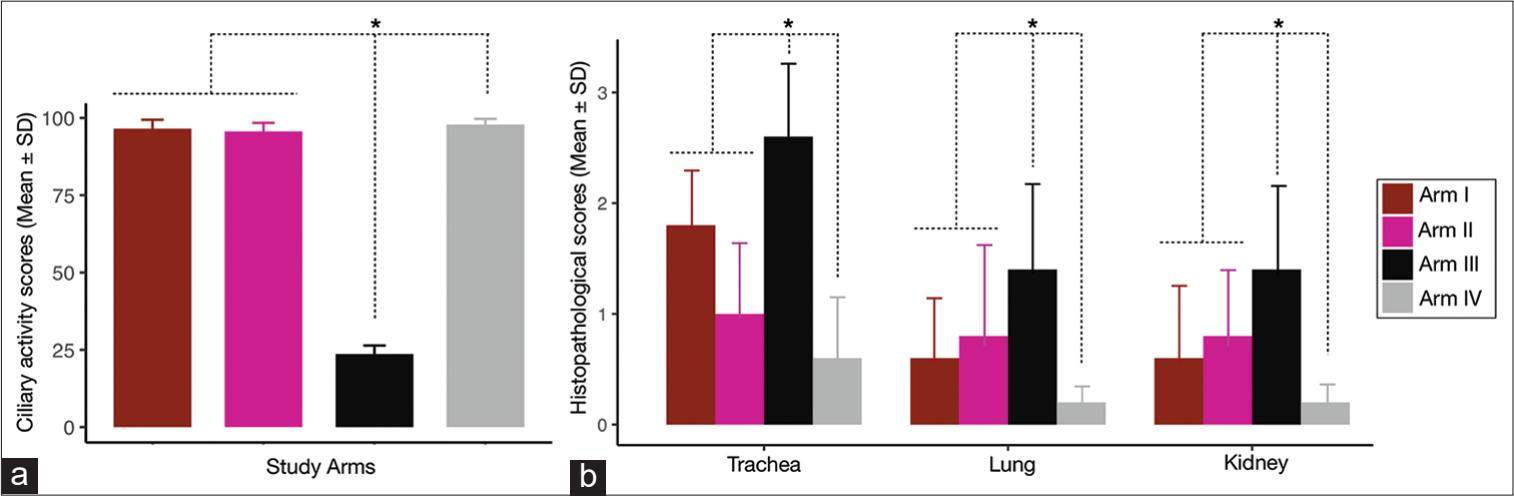
The pathogenic symptoms of chickens measured at 6 dpc of QX-like virulent virus. (a) Cilia-activity; (b) Histopathological scores (*p < 0.05, Kruskal–Wallis tests).

Inhibition of ciliary activity in the trachea was assessed at 6 dpc Arm III exhibited the lowest mean ciliary activity score of 23.64 (SD ± 1.74) with the challenge of local Thai QX-like viruses. The mean scores of ciliary activities were 96.50 (SD ± 1.91) and 95.64 (SD ± 1.77), respectively, in the arms with vaccinated birds, including Arm I (TAbic IB VAR [233A] + H120 vaccines) and Arm II (Ibird [1/96] + H120 vaccine). These values exceeded 50% and 100% of birds were protected (approximately 80%), indicating adequate protection of the vaccines. In addition, the negative control group (Arm IV) displayed a notably high mean score of 97.84 (SD ± 0.89). Significantly different ciliary activity scores (Kruskal–Wallis test, p = 0.05) were observed between Arm III and the other three arms [Figure-2a].

### Histopathological evaluation

Figure-2b presents the results of the histopathological assessment of the tracheas, lungs, and kidneys, along with their scores. The greatest histopathological changes were observed in Arm III (non-vaccinated-challenged birds), with moderately severe changes in the trachea (mean scores: 2.6, SD ± 0.6), and relatively moderate changes in both lungs and kidneys (mean scores: 1.4, SD ± 0.9). On the other hand, tracheal changes ranged from mild to moderate among birds in Arms I and II, with scores ranging from 1.0 (SD ± 0.6) to 1.8 (SD ± 0.5). The lungs and kidneys exhibited mild changes, with scores ranging from 0.6 (SD ± 0.6) to 0.8 (SD ± 0.5). No histopathological changes in the tracheas, lungs, or kidneys were observed in Birds in Arm IV (negative control). Significant differences in histopathological scores were found between arms 3 and 3 (Kruskal–Wallis test, p = 0.05).

## Discussion

Vaccination is essential for preventing the severity of clinical manifestations and mortality associated with IB infections in chickens. Adopting this proactive approach creates a protective barrier that mitigates the impact of IB infection in birds and minimizes economic losses in poultry production systems. Our study revealed the efficacy of heterologous IBV vaccines available in Thailand, including combined 793/B vaccines (TAbic IB VAR [233A] or Ibird (1/96)) and Massachusetts serotype vaccine (H120). Moreover, the absence of IBV detected in the vaccinated birds demonstrated this effectiveness. Furthermore, sustained vigorous ciliary movement and mild histopathological lesions in the trachea, kidneys, and lungs observed in vaccinated birds further underscored the protective outcomes of the vaccines used in this study.

Since the first detection of the QX-like virus in 2009 and its subsequent spread to be a predominant strain in Thailand [15], numerous trials with diverse vaccination programs for poultry protection against the QX-like virus have been conducted [26, 29]. Various commercial vaccines available in Thailand (i.e., QX, 793/B, Mass) were utilized in both homologous (QX vaccine) and heterologous (i.e., QX + B1/Mass/Conn, H120 + B1/Mass/Conn, QX + H120, H120 + 4–91) vaccination approaches. These vaccination programs have shown partial protective effects, such as reducing clinical signs and maintaining ciliary activity. However, these vaccination programs did not provide sufficient protection against Thai QX-like IBV in other aspects regarding IBV detection for re-isolation after challenge. No significant differences were observed between the vaccinated and non-vaccinated groups in these studies. Based on the insights from the previous studies, the present study is the first to apply heterologous IBV vaccines of a combined 793/B vaccine (TAbic IB VAR (233A) or Ibird [1/96]) and the Massachusetts serotype vaccine H120 in Thailand. Our findings highlight the efficacy of this combination of these vaccines in protecting against the local Thai QX-like virus. We employed the same Thai QX-like virus (THA80151 strain) and adhered to the manufacturer’s recommended vaccination schedule and doses, including a heterologous program combining Mass and 793/B vaccines (similar to H120 + 4–91 in one of the studies [26]). Notably, a critical divergence in vaccine efficacy can be attributed to the origin of the experimental birds. Our study utilized SPF chicks raised in controlled, pathogen-free environments, potentially having a distinct immune response compared to broilers used in other studies. Additionally, despite vaccines such as 4–91 and TAbic IB VAR (233A) and Ibird (1/96) belonging to the same serotype 793/B in our research, genetic variations within the spike protein gene of the 793/B serotype of IBV may result in varied impacts on vaccine efficacy, emphasizing the influence of strain-specific genetic diversity [30].

Similarly, the previous studies using a similar vaccination program have highlighted the protective effect in SPF chicks against the QX-like virus. A study by Karimi *et al*. [31] revealed that the combination of Mass (day 1) + 793/B (day 14), challenged with the IBV QX genotype in SPF chickens on day 35, could create 80% protection. Another study applied a vaccination program combining H120-793/B (day 1) + H120 (day 14), challenged with QX-like virus at 35 dpc, and obtained 81% protection [23]. The protective effect can be attributed to a broad-spectrum immune response induced by the combination of these vaccines. Thai QX-like IBV has been identified as a recombinant virus [16], which may result in genetic diversity and reduce the effectiveness of this vaccine. In addition, the current use of live attenuated vaccines may contribute to the evolution of IBV through recombination with other variant genotypes of IBV circulating in Thailand [32]. Therefore, the application of new heterologous vaccines is crucial to address different variants caused by the recombination of Thai QX-like virus.

Although viral culture in embryonated chicken eggs is considered the gold standard for IBV detection [33], PCR-based methods have been widely employed in recent IBV studies due to their rapidity and high sensitivity [34]. Our study utilized RT-PCR and found that IBV shedding in nasal cleft swabs with a challenge dose of 1.5 × 10^4^ EID_50_/bird was observed at 4 dpc, reaching 100% at 6 dpc. Notably, the virus was not detected in samples obtained from kidneys or cloacal swabs until 6 dpc. Respiratory epithelial cells are the primary site for IBV entry and replication, leading to respiratory symptoms and lesions [35]. Therefore, histopathological scores were observed to be higher in the trachea than in other organs, such as the kidneys, lung, and intestine. The previous studies have indicated virus detection in kidneys after 7 dpc with a challenge dose of 10^4^ EID_50_ [26, 29], and in cloacal swabs after 14 dpc with a challenge dose of 10^5^ EID_50_/bird [29, 36]. When comparing histopathological lesion scores in the trachea, kidney, and lung, the efficacy of the two vaccination programs differed across organs. In Arm 1, the kidneys and lungs are better protected, whereas in Arm 2, tracheal protection is more evident. This suggests that the effectiveness of vaccine against the virus differs in different organs. Differences in the role of mucosal and cellular responses and vaccine distribution to different organs may contribute to these variations [37, 38].

Our study investigated various parameters regarding the effectiveness of pathogens and vaccines in protecting SPF chicks against local Thai QX-like IBV strain. This study primarily focused on viral shedding, histopathological changes, ciliary activity, and clinical signs. It is suggested that further investigations should be carried out to broaden our understanding and apply the same vaccine programs to commercial broilers. In addition, we propose examining antibody titers using serological tests and monitoring changes in body weights among commercial broilers. Despite the limitation in sample collection, involving a random subset of birds in each arm, the findings initially showcase the feasibility and preliminary efficacy of two vaccination programs against the local Thai QX-like strain in SPF chick. Therefore, we propose further investigation to assess the efficacy of these vaccination programs when scaled up to a larger population. The aim of this study is to provide insights into the efficacy of the combined 793/B vaccine (TAbic IB VAR [233A] or Ibird [1/96]) and the Massachusetts serotype H120 vaccine in poultry production systems in Thailand.

## Conclusion

This study highlights the critical role of vaccination in preventing the severity of clinical signs and mortality associated with IB infection in chickens. The application of heterologous IBV vaccines available in Thailand, including a combined 793/B vaccine and Massachusetts serotype vaccine, showed significant protective efficacy against the local Thai QX-like virus in chickens. This proactive vaccination strategy creates a protective barrier, helps to mitigate the impact of IB infection on birds, and reduces economic losses in the poultry industry.

## Authors’ Contributions

TT and NC: Conceived and designed the study. TT, DHP, and NC: Conducted the study and drafted and revised the manuscript. TT and DHP: Data analyses. All authors have read, reviewed, and approved the submitted version of the manuscript.
